# Involuntary psychiatric hospitalisation of children and adolescents: a retrospective analysis of Athens public prosecutor’s office for minors records

**DOI:** 10.1007/s00787-025-02821-7

**Published:** 2025-09-15

**Authors:** Christina Anna Stylianidou, Athanasios Douzenis, Ioanna Giannopoulou

**Affiliations:** 1 Medical School, National and Kapodistrian University of Athens, Athens, Greece; 2 2nd Department of Psychiatry, Medical School, National and Kapodistrian University of Athens, Athens, Greece

**Keywords:** Involuntary hospitalisation, Emergency psychiatric evaluation, Minors, Adolescents, Prosecutor’s order

## Abstract

This retrospective study examines emergency involuntary psychiatric admission procedures for minors in Athens (2018–2022), using case records accessed through collaboration between the ‘postgraduate Master’s programme at the Medical School of Athens and the Public Prosecutor’s Office for Minors Athens. Findings indicate a 37.9% increase in involuntary psychiatric examination cases post-pandemic (*p* <.01), though the 20.9% rise in involuntary admissions was not statistically significant (*p* =.140). Analysis of 2019–2021 dataset reveals that most cases handled by the prosecutor involved boys (69.4%) and minors aged ≥ 16 years (54.9%). Involuntary psychiatric examination was ordered in 92.2% of requests and conducted in Child and Adolescent Psychiatry (CAP) Units (54.7%) or Adult Psychiatry (AP) Units (45%). Involuntary admissions occurred in 44.4% of cases, significantly more in AP Units (74.4%) than CAP Units (23.8%). Psychiatric evaluation in an AP Unit (OR = 5.52, *p* =.001), prior contact with mental health services (OR = 2.22, *p* =.016), and older age (OR = 1.32, *p* =.005) were significantly associated with involuntary hospitalisation. Findings highlight the need to expand access to child and adolescent mental health services. Addressing systemic gaps in care could reduce reliance on judicial pathway to care and foster a more preventive and supportive approach to youth mental health.

## Introduction

Involuntary psychiatric hospitalisation of minors remains a complex and contested issue within mental health care. While such measures are sometimes necessary to prevent harm and ensure access to critical psychiatric treatment, they raise ethical, legal, and developmental concerns regarding young people’s rights, autonomy, and long-term mental health outcomes. The prevalence of involuntary psychiatric admission for adolescents differs across Europe that maybe accounted for by the differences in mental health laws, healthcare systems, and legal frameworks [1]. Earlier studies showed 1.6-fold increase in rates of involuntary inpatient treatment in Finland from 16.2% in 1996 to 26.3% in 2003 [2] and decrease from 32.4% in 2004 to 25.7% in 2009 in Germany [3]. A recent study in Germany (Bavaria) found that 24.6% of emergency inpatient admissions for children and adolescents were involuntary [4]. The latest scoping review [5], published in 2024, on the detention of children and adolescents under mental health legislation reported that about one-fifth of psychiatric admissions for minors were involuntary. It further identified that children from minority ethnic communities and those with a history of abuse were more likely to experience involuntary admission, whereas severe mental illness, psychosocial burden, and observable behavioural disturbances constituted the principal grounds for involuntary detention [5].

In Greece, the involuntary mental health assessment procedure is primarily governed by Law 2071/1992 (art. 95 et seq.), which outlines the criteria and steps for the involuntary psychiatric admission of individuals irrespective of age, encompassing both adults and minors. According to article 95 par. 2 of the above-mentioned law, the criteria for involuntary hospitalization are: (I) a. The patient must suffer from a mental disorder, b. The patient must be incapable of assessing what is in the interest of their own health, c. The absence of hospitalization must result in a deterioration of the patient’s health condition, or (II) Hospitalisation of a patient suffering from a mental disorder must be necessary in order to prevent acts of violence either against the patient themselves or against others. It should be noted though that following the enactment of Laws 2619/1998 (Ratification of Oviedo Convention) and 3418/2005 (Code of Medical Ethics), the criteria for involuntary hospitalisation have been partially revised. Specifically, it has been argued that the second set of criteria—namely, that a person be both mentally ill and dangerous—has been implicitly abolished [6]. When the involuntary psychiatric admission procedure concerns minors, the Public Prosecutor’s Office for Minors is the authority responsible for initiating and coordinating the entire process, including, inter alia, the issuance of a legal order for the psychiatric evaluation and/or involuntary hospitalization.

There is a lack of systematic data on the involuntary psychiatric evaluation and hospitalisation of minors in Greece, with most existing studies focusing on adult populations. A study conducted between 2017 and 2020 reported that over 50% of psychiatric admissions in Athens and Thessaloniki were involuntary, compared to 25% in Alexandroupolis, suggesting regional disparities linked to healthcare system organization [7]. Another study in the Athens region documented high involuntary admission rates (60.7%), with psychiatric hospitals exhibiting higher rates (63.1%) than general hospitals (52.8%) [8].

Despite growing concerns about the lack of national statistical data, the revolving-door phenomenon (high re-hospitalisation rates), and insufficient safeguards to protect patient rights [7], research specifically examining psychiatric detention among minors remains scarce. Some insights are provided by Voultsos et al. [9], who analysed involuntary hospitalisation trends in the Department of Child and Adolescent Psychiatry of the General (tertiary level) Hospital “Hippokration” in Thessaloniki (prefecture of Macedonia-Northern Greece) and found a significant increase in prosecutor-ordered admissions between 2005 and 2014, driven mostly by organizational factors rather than clinical necessity. Their findings pointed to inconsistencies in admission criteria, raising concerns about the potential overuse of psychiatric detention among youth.

The current retrospective study covers a consecutive period of 5 years, utilizing data from the Public Prosecutor’s Office for Minors in Athens to address the gap in research on involuntary psychiatric hospitalisation of minors in Greece. It aims to (1) examine longitudinal trends (2018–July 2022) in cases related to the involuntary hospitalisation procedure in minors from pre-Covid to post-COVID period, (2) examine the demographic and clinical characteristics of the minors referred for involuntary psychiatric evaluation or subsequently admitted over a three-year period (2019–2021), and (3) explore recurring patterns in involuntary examination and hospitalisation procedures for minors.

## Materials and methods

In February 2022, a cooperation memorandum was established between the postgraduate Master’s programme at the Medical School of Athens and the Public Prosecutor’s Office for Minors in Athens which issues legal orders for psychiatric examination of minors (aged < 18 years) in most areas of Attica Prefecture. This agreement allowed access to “Psychiatric” case records related to involuntary hospitalisations from January 1, 2018, to July 31, 2022. The case files contained official documents and reports submitted to the Prosecutor’s Office. Extracted data included: demographic characteristics (sex, age, nationality, living arrangements, and legal status), police involvement, identity of the individual requesting the psychiatric evaluation, outcome of the psychiatric examination (involuntary admission or not), care setting (Child and Adolescent Psychiatry or Adult Psychiatry Unit) and discharge diagnosis.

## Results

### Longitudinal trends from pre-Covid to post-COVID period

Between January 1, 2018, and July 31, 2022, the Public Prosecutor’s Office for Minors in Athens handled 663 cases in which a request for hospital admission was submitted. In 92.6% cases (*n* = 614) a prosecutor’s order was issued for an emergency psychiatric examination to determine the necessity of involuntary hospitalisation. Out of the 614 cases, 254 (41.4%) resulted in involuntary admission.

Comparing the pre-COVID period (January 1, 2018– February 29, 2020) with the post-pandemic outbreak period (March 1, 2020– April 30, 2022), the number of cases handled by the Public Prosecutor for Minors increased by 33%, from 270 to 359 cases, a statistically significant change (*p* <.001). Similarly, the number of involuntary psychiatric examination cases rose by 37.9% (*p* <.01). However, while the number of involuntary psychiatric admissions increased by 20.9% (from 110 to 133 cases), this increase was not statistically significant (*p* =.140).

### Demographic and clinical characteristics

The Public Prosecutor’s Office for Minors in Athens processed 461 cases involving 322 minors between January 1, 2019, and December 31, 2021. The demographic and procedural characteristics of these minors and cases are presented in Table 1.

**Table 1:** Cases handled by the Athens Public Prosecutor’s Office: demographic and procedural characteristics (*N* = 461)

Characteristic	*n*	%
Request filed by		
– Relatives	218	47.3
– Residential care staff	140	30.4
–Mental health professionals	15	3.3
–Other/Unknown	88	19.1^a^
Gender		
– Male	320	69.4
–Female	141	30.6
Age ≥ 16 years	253	54.9
Born in Greece	251	54.5
Prior contact with mental health services	377	81.8
Prior contact with involuntary hospitalisation	136	29.5
Emergency legal order granted	425	92.2
Actual assessments conducted	378	88.9^b^
– In Child and Adolescent Psychiatric Units	208	55.0
– In Adult Psychiatric Units	170	45.0
Outcome - Involuntary admission	168	44.4

^a^ Includes 2.8% unknown, ^b^ of the 425 orders for psychiatric examination, ^c^ unaccompanied minors, refugees/asylum seekers, minors in detention or under state protection

Among the total psychiatric evaluations conducted, only 44.4% (168/378) of the cases resulted in the involuntarily admission of the minor. Table 2 summarizes the minors’ demographics, 

care settings, and discharge diagnoses. The mean length of hospital stay was 25.6 days (*SD* = 30.7). Although a downward trend in length of stay was observed from 2019 to 2021, the change was not statistically significant (*p* =.062).

**Table 2:** Involuntary hospitalized minors: demographic characteristics, care settings, length of hospital stay and diagnostic categories assigned at discharge (*N* = 168)

Characteristic	*n*	%
Setting		
– Child and Adolescent Inpatient Psychiatric Unit (CAIP)	40	23.8
– Adult Psychiatric Inpatient Unit (AIP)	125	74.4
– Both CAIP & AIP	3	1.8
Gender: Male	123	73.2
Age ≥ 16 years	136	81.0
Lived with family prior to admission	94	56.0
Born in Greece	88	52.4
Special legal status ^a^	67	39.9
ICD-10 Diagnostic category at discharge ^b^		
– F10–19 Psychoactive substance use	12	7.9
– F20–29 Psychotic disorders	24	15.8
– F84/F70 Pervasive developmental disorders/intellectual disability	13	8.6
– F40–49 Anxiety disorders	23	15.1
– F30–39 Affective disorders	19	12.5
– F90–98 Behavioral & emotional disorders	20	13.2
– F60–69 Personality disorders	13	8.6
– Comorbid conditions	22	14.5
– Other diagnostic codes	6	3.9
Length of hospital stay (Mean ± SD in days)	25.6 ± 30.7	
– 2019	32.5 ± 34.2	
– 2020	28.1 ± 35.0	
– 2021	19.7 ± 24.7	

^a^ unaccompanied minors, refugees/asylum seekers, minors in detention or under state protection; ^b^ based on 152 cases with available discharge data

A binary logistic regression analysis was conducted to examine factors associated with involuntary admission (dependent binary outcome), considering the following independent variables: sex (female/male), age, special legal status (yes/no), previous contact with mental health professionals (yes/no), Mental Health Unit conducting the evaluation (AP/CAP). The model was statistically significant, *χ*²(5) = 108.674, *p* <.001, explaining 34.4% of the variance (Nagelkerke R²=0.344). Results showed that minors were approximately five times more likely to be involuntarily hospitalised when their psychiatric evaluation was conducted in AP rather than CAP Unit, B = 1.709, SE = 0.319, Wald = 28.661, *p* <.001, OR = 5.521 (95%CI 2.954, 10.321). Previous contact with mental health professionals doubled the likelihood of involuntary hospitalisation, B = 0.799, SE = 0.333, Wald = 5.752, *p* =.016, OR = 2.223 (95%CI 1.157, 4.270). Furthermore, each additional year of age increased the probability of involuntary hospitalisation by 31.8%, B = 0.276, SE = 0.099, Wald = 7.757, *p* =.005, OR = 1.318 (95%CI 1.127, 1.657). No statistically significant associations were found between involuntary hospitalisation and sex (*p* =.158), or special legal status (*p* =.100).

### Recurring patterns related to involuntary hospitalisation procedures

Analysis of 2019–2021 case files revealed several recurrent patterns related to involuntary hospitalisation procedures for minors that raise concern. These included: (a) issuing involuntary examination orders sometimes without sufficient supporting evidence, (b) brief and sometimes unjustified medical evaluations following emergency psychiatric assessment, (c) explicit reports of unavailability of beds in CAP Units, leading, among others, to the admission to an AIP Unit, (d) repeated initiation of the legal procedure for involuntary hospitalisation within less than one month of a previous psychiatric evaluation or hospitalisation, (e) hospitalisation decisions based on non-clinical and administrative considerations, such as lack of alternative care options (e.g. securing placements for unaccompanied minors within the care system, facilitating their transfer to alternative accommodation facilities), rather than being driven solely by medical necessity, (f) police involvement in the vast majority of the cases, at various stages of the procedure, particularly during the transfer of the minor for examination.

## Discussion

The present study found a 33% rise in cases handled by Athens’ Public Prosecutor’s Office for Minors when comparing 1 January 2019–29 February 2020 (pre-COVID) with 1 March 2020–31 December 2021 (post-outbreak). This surge mirrors a Europe-wide survey of 110 heads of child and adolescent psychiatry departments reporting a marked increase in assessment requests by early 2021 [10] and aligns with moderate elevations in depressive and anxiety symptoms among Greek youth [11]. Three interrelated factors may underlie the post-outbreak increase in the use of involuntary psychiatric examination procedure for minors. The pandemic acted as a prolonged psychosocial stressor that exacerbated pre-existing vulnerabilities in line with a stress–diathesis model [12]. In addition, lockdown measures and concomitant economic hardship intensified intrafamilial conflict and undermined informal support networks [13]. Finally, years of austerity-driven underfunding had already weakened Greece’s public mental health and child-welfare systems [14, 15]. Collectively, these pressures seem to have prompted more families and legal guardians to seek judicial referral as a means of securing psychiatric care for youth. Involuntary psychiatric examination cases increased by 37.9%, suggesting unmet demand amid constrained resources and limited access to outpatient child and adolescent mental health services (3.6 per 100 000 under-18s) [16]. In contrast, involuntary hospitalisations remained unchanged (*p* =.140), reflecting the persistent shortage of inpatient beds for young people (3.01 beds per 100 000 under-18s nationwide, with only 10 beds in Athens for adolescents aged ≥ 16) [16, 17].

Data analysis reveals a striking pattern: over 92% of involuntary admission requests are accepted by the prosecutor. Yet fewer than half of minors evaluated under this procedure are ultimately hospitalised (44.4% in 2019–2021; 46.4% in 2018–July 2022). By contrast, adult studies show involuntary hospitalisation rates of 96.9% (in Attica) and 87.5% (nationwide) following involuntary evaluations [18, 19], highlighting the different application of Law 2071/1992 to adults versus minors. The high approval yet low hospitalisation rates for minors, contrasting sharply with adult cohorts, may suggest that prosecutors apply a different threshold for initiating involuntary procedures in case of minors. This practice possibly aims at safeguarding vulnerable youth and securing immediate assessment and likely stems from requests of concerned families or care staff and from the scarcity of community-based crisis alternatives.

Analysis of the 2019–2021 dataset of involuntary hospitalised youth revealed a predominance of boys (73.2%) and adolescents aged ≥ 16 years (81%). Notably, minors with prior contact with mental health services were over twice as likely to be involuntarily hospitalised. Each additional year of age increased the likelihood of admission by nearly 32%. Taken together, these findings may indicate systemic shortcomings in early identification and stepped-care pathways for high‐risk minors, underscoring the need for developing age- and gender-sensitive outreach [20].

One particularly concerning finding is the widespread use of AP Units for evaluating and admitting minors, despite the Special Monitoring Committee for the Protection of the Rights of Persons with Mental Disorders [21] recommending that such placements be limited to cases of absolute necessity. Nearly half of legally ordered emergency evaluations and almost three-quarters of involuntary admissions took place in AP settings - half of these in standalone mental health hospitals such as Dafni and Dromokaiteio, which directly contravenes the Committee’s guidelines. Compared with just 5.2% of under-18 hospitalisations in Ireland [22] and 22.6% in Ontario, Canada [23], Greece’s high rate of youth compulsory admissions to AIP Units likely reflects bed shortages in CAP Units (3.01 beds per 100 000 under-18s nationwide, with only 10 beds in Athens for adolescents aged ≥ 16) [16, 17] and raises legal, ethical and clinical concerns about admitting minors to adult wards, where care models may not align with developmental needs and exposure to older patients with severe psychopathology can pose additional psychological, safety and social risks.

The significantly higher likelihood of involuntary hospitalisation following evaluation in AP Units (OR = 5.5, *p* =.001) suggests potential differences in assessment approaches or institutional policies. The diversity of discharge diagnoses among involuntarily hospitalised minors underscores the need to expand access to specialised, age-appropriate psychiatric services that better address adolescent mental health needs.

Although special legal status (e.g., unaccompanied minors, those under state protection or in detention) was not significantly associated with involuntary hospitalisation in our regression model (*p* =.100), these youths accounted for 40% of all compulsory admissions despite comprising only a small proportion (approximately single digit percentage) of Greece’s under-18 population. This marked disproportionality may suggest that legal procedures may serve as a compensatory measure for gaps in mental health and psychosocial support services for this vulnerable group [24]. Consistent with Giannopoulou et al. [25], 42% of emergency psychiatric evaluations in their cohort were activated through prosecutorial legal orders—often at the request of shelter staff. In the same study, 77% of unaccompanied refugee minors assessed were deemed not in need of hospitalisation, underscoring concerns about the appropriateness of psychiatric intervention for this population.

The review of prosecutor case files from 2019 to 2021 uncovers a procedural pathway that sometimes substitutes administrative expediency for clinical rigour. Orders for involuntary examination are occasionally issued on minimal evidence; psychiatric assessments can be cursory or prompted by non-clinical needs; and frequent CAP bed shortages divert youth into adult wards. Rapid re-referrals within weeks, coupled with routine police transfers, further suggest that the system at times functions more as a crisis-management circuit than as a therapeutic safety net. These recurrent patterns highlight gaps in legal safeguards, bed capacity, and community alternatives, and underscore the need for targeted measures to strengthen clinical gatekeeping, expand child-specific resources, and embed clear, evidence-based thresholds into every step of the involuntary hospitalisation process.

While the study provides valuable insights into referral patterns for involuntary psychiatric hospitalization procedures in the case of minors, its interpretative scope is limited by methodological and contextual constraints. The retrospective design, based solely on administrative case files from the Public Prosecutor’s Office for Minors in Athens, lacks linkage to medical records, omitting essential clinical data such as symptom severity, treatment history, risk assessments, attempts at less restrictive interventions and their outcomes. Due to these data gaps, it is not possible to draw conclusions about whether prosecutorial referrals adhered to best clinical practices or were justified under the legal framework governing psychiatric detention. The study’s geographic focus on Athens may limit the generalisability to other regions with different mental health infrastructures. Finally, the absence of follow-up data further restricts understanding of long-term outcomes, including recovery, reintegration, and future service use.

## Conclusion and future directions

This retrospective study highlights key procedural and systemic limitations in the application of involuntary hospitalisation for minors in Athens under Law 2071/1992. Despite high prosecutorial approval rates for psychiatric examination orders, fewer than half of the evaluated minors are ultimately admitted, indicating discrepancy between legal action and clinical necessity. The frequent use of adult psychiatric units, compounded by repeated referrals within short timeframes and routine police involvement, suggest that the use of legal procedural practice is shaped largely by crisis-management pressures rather than structured, developmentally appropriate mental health care.

To improve the alignment between clinical need and legal intervention, future strategies should prioritise the adoption of brief, age-specific risk-assessment tools at the referral stage, enabling more informed and clinically grounded decisions. Strengthening child and adolescent mental health services, including increasing bed capacity and the deployment of mobile or community-based crisis teams, would help reduce reliance on adult units and judicial pathways. Establishing clearly defined legal and clinical thresholds for involuntary evaluation, coupled with expedited review procedures, could enhance procedural fairness and better protect minors’ rights. Furthermore, linking prosecutorial and healthcare datasets, alongside implementing structured audits of Emergency Department visits for acute mental health concerns in youth, would offer a robust foundation for outcome monitoring, resource planning, and future policy refinement.

## Data Availability

The original contributions presented in this study are included in this article. Further inquiries can be directed to the corresponding author.
